# Assessing user preferences for design characteristics of oral dissolvable strips for pediatric HIV medication: a qualitative study

**DOI:** 10.1186/s12913-023-10078-6

**Published:** 2023-10-16

**Authors:** Catherine Wexler, May Maloba, Michala Sliefert, Shadrack Babu, Nicodemus Maosa, Edward Maliski, Zachary Nicolay, Frederick Were, Yvonne Mbithi, George Mugendi, Gregory Thomas, Harshdeep Acharya, Sarah Finocchario-Kessler

**Affiliations:** 1 Department of Family Medicine, University of Kansas Medical Center, Kansas City, KS USA; 2 Global Health Innovations, Nairobi, Kenya; 3 School of Medicine, University of Kansas Medical Center, Kansas City, KS USA; 4 Oak Therapeutics, Lawrence, KS USA; 5 University of Nairobi, Nairobi, Kenya; 6 School of Architecture and Design, University of Kansas, Lawrence, KS USA

**Keywords:** HIV, Pediatric antiretroviral therapy, Antiretroviral therapy adherence, Kenya, Qualitative research, User-centered design

## Abstract

**Background:**

Current infant antiretroviral therapy formulations pose barriers to daily adherence due to complex weight-based dosing, conspicuous preparation, and poor palatability. These adherence barriers jeopardize adherence, making patients vulnerable to virologic failure, development of drug resistance, and preventable mortality. Our team has previously established proof-of-principle for multi-drug oral dissolvable strips as alternative pediatric antiretroviral formulations with the potential to overcome these challenges and improve pediatric ART adherence and outcomes. The objective of this study was to assess caregiver and provider preferences for oral dissolvable strips and its packaging to inform its development.

**Methods:**

Guided by concepts of user-centered design, we conducted key informant interviews with 30 HIV care providers and focus group discussions targeting caregivers of children < 10 years of age living with HIV at 3 Kenyan hospitals. Key informant interviews and focus group discussions were audio recorded, translated/transcribed verbatim, and hand coded for a-priori and emergent themes.

**Results:**

A total of 30 providers and 72 caregivers (caring for 83 children, aged 5 months to 18 years) participated in the study. Caregivers and providers expressed a strong desire for an easier way to administer medication, especially among children too young to swallow tablets whole, and expressed enthusiasm around the idea of oral dissolvable strips. Key preferences included a pleasant taste; one strip per dose; small size with rapid dissolution; clear markings and instructions; and no special storage requirements. For packaging, stakeholders preferred individually wrapped strips within a dispenser. The individual packaging should be durable, waterproof, and easy to dispose of in communal spaces. They should also be easy to open, with clear indications where to open. The packaging holding the strips should be durable, re-usable, accommodating of various refill frequencies, and easy to use for children as young as 6.

**Discussion:**

The concept of oral dissolvable strips was highly acceptable to caregivers of children living with HIV and HIV care providers. By engaging stakeholders in an iterative design process starting from the early phases of design and development, we will maximize the likelihood of developing a product that is acceptable to the caregiver and infant, therefore leading to sustainable adherence.

**Supplementary Information:**

The online version contains supplementary material available at 10.1186/s12913-023-10078-6.

## Background

Global and national investments in prevention of mother to child transmission of HIV (PMTCT) services have contributed to an approximately 60% decline in the number of new HIV infections among children in the last decade [1]. However, there are still an estimated 1.02 million children under the age of 10 living with HIV, globally, with an additional 160,000 new infections annually among children less than 10 [2]. In Kenya, there are approximately 83,000 children living with HIV [3]. These children will require lifelong antiretroviral therapy (ART), starting at the time of diagnosis.

Critical barriers to the provision of and adherence to pediatric ART contribute to only about 50% of children living with HIV on treatment achieving viral suppression in Kenya, compared to 72% of adults [4]. Furthermore, high rates of loss to follow up – up to 34% by 36 months – are observed among children on ART [5]. Regimen related barriers to pediatric treatment include lack of appropriate formulations for recommended drugs, complexity of pediatric dosing (frequently changing dosage), and poor palatability of available formulations. Syrups are unpalatable and bitter causing children to spit or vomit them, require cold storage which is unavailable in low resource and/or rural settings, difficult to measure accurately, and cumbersome to carry from the clinic to the home which puts caregivers at risk of unintentional status disclosure. Tablets formulated for adults can be used for children but need to be cut to correspond to the child’s weight, are often too large for young children to swallow easily even when cut, are difficult to crush, do not dissolve well in liquid, and have a bitter flavor when crushed that causes children to spit or vomit them [6, 7]. Furthermore, pediatric ART adherence is impacted by characteristics of both the child (developmental stage, neurodevelopment, knowledge of HIV status) and by caregiver/family characteristics (caregiver permanence and relationship with child, caregiver health and psychosocial function, poverty), complicating pediatric adherence [8]. Other individual and system-level challenges to pediatric ART adherence include forgetting to take, depression, stigma, long distance to clinics, and frequent supply stockouts [9].

Oral dissolvable strips (ODS) are an alternative drug delivery method that can simplify dosing and administration and improve palatability. ODS have generated interest for other pediatric formulations such as topiramate, ondansetron, sennosides, and diphenhydramine/phenylephrine, [10, 11] and previous studies have established proof of principle for multi-drug ODS [12]. ODS can be adhered on the cheek, tongue or palate allowing for rapid oral dissolution in saliva and absorption, reducing the likelihood of spitting out partial or complete dose. To mask the bitter taste associated with some antiretrovirals (ARVs), flavors can be added to the ODS to give it a pleasant taste. Our team has developed and assessed in vitro bioavailability for an ODS containing high dose, multidrug ARVs suitable for pediatric HIV prophylaxis according to Kenya’s National Guidelines [13]. All ODS characteristics evaluated (encapsulation, degradation, oral dispersion, in vitro bioavailability, stability) met or exceeded targeted criteria, indicating non-inferiority of ODS (AZT + NVP) compared to bulk drug standards [12]. A current SBIR (1R43AI170237) aims to use a modified technology that can support ODS loading of up to 450 mg of active pharmaceutical ingredients to develop an ODS containing therapeutic dose of pediatric ART.

The Conceptual Framework for Pediatric Adherence to HIV ART illustrates the influence of child, caregiver, regimen, and societal characteristics on pediatric ART adherence [8]. Here, we focus on regimen-related characteristics. Pharmaceutical design –including product characteristics and use parameters–can impact acceptability, use, and adherence of pharmaceutical products [14–16]. Implementation research with end-users beginning at the initial development phase is key to successful uptake and utilization of any new product, and it can avoid wasted investment in innovations that are not widely adopted. However, the preferences of patients and caregivers– who are most affected by new therapies - are often overlooked in the design and development of new medical technologies, [17, 18] spurring a recent focus on engaging patients throughout the continuum of medical product development to ensure patients’ experiences, needs, and priorities are understood and incorporated into drug development and evaluation [18–20].

The objective of this study was to assess user (caregiver and provider) preferences for ODS and its packaging to inform ODS development. This parallel effort of assessing user preferences for ODS characteristics (R21HD105534) will maximize the likelihood of acceptability and enhance the ultimate impact of this innovative pharmaceutical product.

## Methods

### Study overview

This study was part of a larger effort (Grant Number R21HD105534) to use a human-centered design approach develop ODS for pediatric ARV prophylaxis and ART in Kenya. Our three-phased approach included steps to (1) use qualitative methods to assess initial stakeholder preferences for ODS and packaging, (2) develop ODS and packaging prototypes based on phase 1 results, and (3) seek final feedback on the developed prototypes to finalize strip and dispenser design. Results of phase 1 are presented in this manuscript.

### Phase 1 procedures

The study was implemented at three hospitals in Siaya (n = 2) and Mombasa (n = 1) counties of Kenya from February to April of 2022. The study hospitals were selected in Western and Coastal regions of the country, representing cultural and religious diversity that could influence practices and behaviors. Eligible participants included caregivers of children living with HIV under 10 years of age and HIV care providers at study hospitals.

#### Caregiver focus groups eligibility

Caregivers were eligible for inclusion in this study if they cared for at least one child living with HIV 10 years of age or younger and were able to provide informed consent.

Caregivers participated in focus groups consisting of 8–12 participants. At each hospital, four groups of caregivers were targeted: caregivers of children living with HIV < 1 year, with children 1–2 years of age, with children 2–5 years of age, and with children 6–10 years of age. If patient volumes at study hospitals were not adequate to obtain 8–12 participants per focus group, caregiver groups were combined to achieve the minimum participant number. Different caregiver types (mothers, fathers, aunts/uncles, grandparents, etc.), of varying HIV statuses (HIV+, HIV-, unknown HIV status) and various ages were purposefully sampled. Mentor mothers (mothers living with HIV who had gone through PMTCT/early infant diagnosis (EID) services at the hospital) reached out to eligible participants via phone or at their next appointment.

#### Provider interview eligibility

A total of 10 HIV care providers from various departments (maternal and child health [MCH], comprehensive care centers [CCC], outpatient departments [OPD], Pharmacy) and with various roles (clinical officer, nurse, pharmacist, mentor mother) were targeted at each study hospitals, for a total target of 30 providers interviewed across the three study hospitals. The in-country PI (MM), who is familiar with clinical operations at each study hospital, worked with hospital administration to select provider interviewees.

#### Enrollment and consent

Eligible participants who wanted to participate were asked to come to the hospital for the scheduled FGD. Prior to participating in the study, all caregivers were required to provide written informed consent. Study procedures were approved by the Institutional Review Boards at the University of Kansas Medical Center (STUDY00147024) and the University of Nairobi (P457/06/2021).

#### KII and FGD methods

Immediately following the informed consent interview, the research assistant conducted a quantitative survey with all participants who consented to study participation. This quantitative survey assessed participant demographics, clinical care history, and perceived barriers to pediatric ART administration.

Semi-structured KII and FGD guides were developed based on a priori areas of interest identified from the literature and from prior experience of the research team and ODS development team. The instruments were pilot tested with Kenyan study staff and co-investigators to ensure straightforward language and clarity. Key topics included challenges to standard of care pediatric ART administration, initial perceptions of ODS, preferred characteristics of ODS, and preferred characteristics of ODS packaging and dispensers, see supplementary materials 1 and 2 for FGD and KII guides. FGD were conducted in the participants’ language(s) of choice (Luo, Swahili, English) and KII were conducted in English. Interviews lasted approximately 45 min and FGD lasted approximately 90 min.

All interviews and FGD were conducted by two trained research assistants (authors SB (male), GO (female)) in a private room at the hospital. Participants did not have a prior relationship with interviewers and were told that interviewers were part of a team working to develop ODS for pediatric ART. Prior to the study, facilitators received a general training on qualitative research methods and a study-specific training on the research tools for this study.

#### Analysis

All KII and FGD were audio recorded and translated/transcribed verbatim. Transcripts were not returned to participants for comment or review. Three analysts (MS, CW, HA) hand-coded the transcripts for *a priori* themes related to standard of care pediatric ART administration, perceptions of ODS, and preferred characteristics of ODS and packaging, see supplementary material 3 for code tree. All coders coded two transcripts of each type (provider and caregiver) together to ensure consistency of coding. Afterwards, analysts met weekly to develop and refine a codebook through iterative consensus building. Disagreements between coding were resolved by the senior author. Once coding was complete, analysts developed memos to summarize each code. Exemplars for each code were noted as well as the frequency and distribution of themes within the larger topic areas.

Methods were carried out and reported according to the Consolidated criteria for reporting qualitative research, see supplementary material 4 for the COREQ checklist.

## Results

In total, we conducted 9 FGD and 30 KII. In total, 72 caregivers and 30 providers participated, see Tables 1 and 2 for caregiver and provider characteristics. The 72 caregivers cared for a total of 83 children, ranging in age from 5 months to 18 years. The caregiver of the 18-year-old also cared for two younger children living with HIV and, therefore, met the eligibility criteria of the study. This caregiver talked about all her children during interviews and, thus, we opted to include the 18-year-old in the summary of caregiver characteristics.

**Table 1:** Caregiver Characteristics

	N	%
Gender		
Females	60	83.3%
Relationship to Child		
Grandparent	14	19.7%
Aunt	8	11.3%
Parent	43	60.6%
Other	1	1.4%
Sibling	3	4.2%
Step mother	2	2.8%
Number of HIV + Children in Care		
1	61	84.7%
2	10	13.9%
3	1	1.4%
Disclosure		
Disclosed to anyone	69	95.8%
“I am the only one who gives the child his/her ART”		
TRUE	23	31.9%
Weekly household income		
< 500	32	44.4%
500–750	18	25.0%
750–1000	9	12.5%
1000–2500	9	12.5%
> 2500	4	5.6%
Level of education		
No school	2	2.8%
Some Primary	24	33.3%
Completed Primary	19	26.4%
Some Secondary	11	15.3%
Completed Secondary	13	18.1%
Some college/university	3	4.1%
Caregiver HIV Status		
HIV+	55	76.4%
HIV-	16	22.2%
Unknown	1	1.4%

**Table 2:** Provider Characteristics

Sex		
Female	19	63.3%
Provider role		
Mentor Mother	3	10.0%
Pharmaceutical Technologist	3	10.0%
CHV	3	10.0%
Nurse	7	23.3%
Clinical Officer	6	20.0%
Administrator	3	10.0%
Other	5	16.7%

### Initial perceptions of ODS

Caregivers and providers expressed enthusiasm about the concept of ODS for pediatric ART administration. Both caregivers and providers agreed that ODS could overcome many challenges that caregivers faced in the provision of pediatric ART. The greatest perceived benefit driving enthusiasm was the ease of administration – compared to current processes – especially if taste masking was successful. Other perceived benefits included more discreet administration, easier portability for administration during times of travel, and discreet storage (as compared to syrups).

*The ODS concept is incredibly good. It can be an alternative formulation…It just looks nice to me. It might be able to help improve pediatric ART adherence and overcome the challenges they are having in terms of bulkiness, the taste. We are really looking forward to having such an implementation, so that pediatrics can be able to adhere to ART. [Hosp1_KII2]*

*“if possible we could immediately start using the ODS because as you have explained it will be easy, the fact that it dissolves in saliva is great so we don’t have to struggle with the child or have to look for porridge.” (Hosp3_FGD2_ID8)*.

### Preferred characteristics of ODS

Participants described the key characteristics of ODS that would facilitate acceptance at both the individual and hospital levels. Key characteristics in approximate order of importance included: taste, size, dissolution, frequency, markings, and other characteristics (including color, smell, shape, storage, texture, dose size, instructions).

#### Taste

Caregivers and providers overwhelmingly agreed that taste was the most important characteristic of ODS. All agreed that ODS should have a “*sugary taste and sweet taste”* (Hosp3_FGD1_ID6) to overcome current challenges related to child refusal of medication, anxiety around medication administration, and vomiting after medication administration due to the bitter taste of current formulations. Given a good taste, caregivers and providers were willing to compromise on other preferred characteristics.

*I think to me the one that should be given priority is taste. Taste matters because even if the drug is white but the taste is sweet even if it takes 10 min in the mouth so long as it is sweet the child will not spit it nor vomit it. But if the shape is okay and the color is great too but it is not sweet, even if it dissolves fast, they will try and spit it or vomit it out. So, for me taste matters*. *(Hosp3_KII9)*

While a sweet and child-friendly taste was uniformly agreed upon, there was less consensus on what specific flavor the strips should be. It was stated that flavoring should represent locally available flavors. Table 3 represents the number of times various flavors were recommended by caregivers and providers.

**Table 3:** Preferences for ODS flavors

Flavor	Provider Frequency	Caregiver Frequency	Total frequency
Strawberry	23	8	31
Orange	9	11	20
Vanilla	8	1	9
chocolate	3	4	7
Pineapple	2	4	6
Banana	3	3	6
Mango	4	0	4
Passion Fruit	4	0	4
Honey	0	1	1

*For example, taste of strawberry, a taste of an orange, a taste of a mango, a taste of a pineapple, a taste of a passion fruit: the things they commonly eat around in the households and locally available in most localities, not complex flavors that don’t exist in our setup. (Hosp3_KII10)*

Some providers expressed concerns that given the very bitter taste of the drug, adequate taste masking would be challenging to achieve. In the absence of adequate taste masking, the potential benefits of ODS were significantly minimized and providers would prefer to continue prescribing standard regimens.

*“It can have the sweet flavors- chocolate, honey etc. But remember not all drugs will work with it. Some will maintain the bitter taste even after dissolving in the mouth. It will just be better that we continue administering Kaletra using syringes, instead of infusing it in ODS with its bitterness. (Hosp1_FGD2_ID5)*

#### Size and shape

Size was considered a very important characteristic by most providers and caregivers. In general, they felt that the strips should be as small as possible; however, a quantifiable size was difficult to nail down, and some stated that acceptable strip size was dependent on child age. The smaller of the ODS sample strips displayed to participants was 2.7 cm x 1.7 cm and they felt that this size was acceptable but would prefer an even smaller sample. They felt that anything bigger would be unacceptable. Furthermore, some providers noted that thickness of the strip should also be considered, with thinner strips preferred over thicker strips. Providers feared that large strip sizes would make it difficult to administer the drugs to the children.

*Something closer to this [strip, 2.7 cm x 1.7 cm], yeah, if we can maintain it something not bigger than this, that will be very okay, I’m looking at the small babies and just trying to see if you were to, you don’t want something that if you put on the tongue, part of it is sticking out. (Hosp3_KII4)*

Strip shape was noted amongst the least important characteristic by both providers and caregivers. Circle, rectangle, and square were the most mentioned shapes, but no one voiced a strong opinion, and no shape came out as a clear choice.

#### Dissolution

Nearly all providers and caregivers believed the strip should be dissolved as quickly as possible. Most providers recommended a dissolution time ranging from 30 s to 1 min, while caregivers preferred less than 5 s. Most believed that a shorter dissolution period would lead to less likelihood of the child spitting out the drug, decreased anxiety on the caregiver who is supervising, and decreased opportunities for others to notice the administration of the medication.

*If you have something that would take just a minute or so, that would be fantastic, so not something that a baby would be having in the mouth for a long time. So that if you give a baby and you are standing and monitoring the baby for a minute or so, that thing should dissolve. (Hosp3_KII4)*

While, generally, most agreed that dissolution should take as little time as possible, a few discussed how a longer dissolution time would be acceptable – and perhaps even preferable – assuming that the strip was palatable to the child and stayed sweet the entire time that the strip remained in the mouth.

*If the taste is okay the one or two minutes is okay, as long as they still have the sweet taste in their mouth, but it should not stay for long.(Hosp3_FGD3_ID4)*

#### Number of strips

In general, participants expressed a strong desire for a single strip to correspond with a single dose without the need of cutting strips or adding strips for additional dosing. Thus, they recommended strips for each weight category so that as children grew and moved into new weight/dosing categories, they would remain with a single strip per administration.

*we make a special strip based on that weight, where one is designed for a weight range… that will be the best way to go. I think that will be easy for the people dispensing, and for the mothers to administer. (Hosp1_KII9)*

Providers worried about the dangers of under-dosing and overdosing if individual strips for each age group were not able to be developed. They also discussed how the need to administer multiple strips at a time would be a deal breaker for prescribing the drug. While 5 was the number of strips mentioned, this seemed to be more of an example than a hard cut off and there was a strong desire across multiple providers to have a single strip developed for each age category.

*Then also the quantity to give. If it’s a lot I will prefer the current regiment i.e., if it require them to take…let’s say five ODS strips in the morning, and five in the evening. I would consider the current regiment. (Hosp1_KII5)*

#### Storage and shelf stability

Participants stressed the importance of a product that could withstand various temperatures and did not require special storage conditions. Providers discussed how, ideally, ODS could be stored in cupboards or purses at normal temperatures without degrading. Especially at the Coast, which is known for its high temperature and humidity, providers expressed concerns that with improper storage or packaging, the environmental conditions would cause the strip to dissolve outside of the patient’s mouth.

*Something that is sustainable, and can withstand climatic changes, not like other drug types that when exposed to cold, they get sticky, and when exposed to heat, they melt. We want it to maintain its original shape and formula. (Hosp2_KII7)*

Given variable refill frequencies (ranging from 2 weeks to 3 months, depending on various patient and facility factors), it is important that ODS remain shelf stable for enough time that providers can prescribe up to 3 months of medication at a time, without it expiring.

*If I am given five packets of these that is 50 strips, I can keep these even for a month and it will still be in good shape. (Hosp2_KII4)*

#### Marking and instructions

Most providers and participants agreed that drug marking was essential to identify the ODS. Most stated that markings should include medication name, dosage, and expiry date. Having this labeling will help patients provide the correct medication and dosage to their child, especially in the context of needing to provide multiple drugs to his/her child(ren), and would also simplify dosing and adherence monitoring for the provider.

*That’s a must have for every drug. Labels and expiry date are very important. (Hosp1_FGD2_ID5)*

*Of course, I would want the markings added including the strip contents, dosage, etc., so that even if a mother is carrying a remainder of an ODS i.e., if she has travelled, and is only left with one, she can still go to the nearest facility, show it to them and they will quickly know what it is. (Hosp2_KII7)*

There was no clear consensus over whether these markings should be directly on the strip or on the packaging. Arguments for having it on the strip included confidentiality (less likely for others to see an opened strip than for them to see the packaging). Arguments for having it on the packaging included concerns about the ingredients used for label being safe for infant/child consumptions and simplicity for providers who will not be opening each individual strip. Participants felt that concerns about confidentiality with labeled packaging could be mitigated by carefully designed labels.

*So maybe mark it on the strips, but the outside package …….because you see… even your neighbor can ask; “let me see” and they’ll know this is 3TC (Hosp1_KII10)*.

*I would prefer the writings on the package because it will be a packed thing. It might interfere with the medicine … I just don’t like the writing on the drug*. *(Hosp3_FGD2_ID8)*

Providers expressed the importance of both counseling caregivers and patients on the correct way to administer the ODS, as well as clear instructions on dosing and administration included with the ODS for them to refer to at home. Topics that the instructions should touch upon include the need to wash and dry hands prior to administration, dosing amounts, process of opening and dispensing the ODS, and ODS adhere to the child’s palate. A few providers suggested that instructions be visual drawings – rather than textual. This would reduce concerns related to low literacy and use of local languages.

*The instruction should come out clearly for everyone i.e., for the care givers, and the health care workers: All the important information should be readily available on the wrapping. ( Hosp2*_*KII7)*

#### Other characteristics

Less frequently mentioned preferences for the ODS included that: (1) ODS should not come with any food/beverage restrictions, (2) ingredients of the strip be free from any allergens (eggs, gelatin, etc.), (3) sweet smell, and (4) non-sticky texture.

#### Packaging

Packaging for ODS was mostly discussed as an individually packaged strip within a “dispenser” to hold and distribute the strips. Key considerations for packaging included: ease of use (but also balancing considerations for child proofing), size (which had implications for refill frequency, discretion, and portability), material (with implications for medication protection and wrapper disposal), and cost. Participants talked of how these key considerations could be designed to be both functional and non-stigmatizing.

#### Ease of use

While a few caregivers and providers liked the novelty of a tape-like dispenser, they mostly agreed that simplicity and ease-of-use should take priority over novelty and innovation. Caregivers felt especially strongly that the process of measuring and dispensing the medication should be as streamlined as possible so that other caregivers – sometimes children as young as 6 - could dispense the medication without error.

*This first one [tape like dispenser] might be easier for me because I have already been shown explained on how to use it. I’m imagining at home, where I’m not around and the person, or the child supposed to administer the drug, doesn’t know how to operate it; it might bring issues. But for the pouch, it is easy to use because; the ODS is just in a packet, so you’ll only open, pick the strip, and close the pouch. Yes, the first is good. I would really love to pick it, but I can not when I think of the other users. (Hosp1_FGD1_ID5)*

Caregivers and providers unanimously preferred a dispenser that distributed only a single dose that corresponded to the child’s weight band at a time, rather than a tape-like dispenser where caregivers could unroll the needed length of strip based on the child’s dosage, cut the strip, and then reseal the dispenser. Reasons for this preference included: (1) concerns about hygiene, (2) concerns about mis-dosing, (3) concerns about wasted medications, and (4) concerns about heat/humidity if strips are not individually packaged. While providers preferred having single strips per serving, if half strips were needed, they recommended serration and clear marking on the strip where it would need to be cut to ensure correct dosing.

*As for me, I would advocate for single strips, and not the tape like ones. This is because, the tape like ones, will require mothers to be very careful and observant about their hygiene. Or else, we shall experience situations where care givers pull opened a long ODS strip, cut the required piece, and then fold and squeeze back the remainder i.e. after touching it all over. So, it will just be nice to pack single stripes per age and weight of the infant. (Hosp1_KII1)*

Providers and caregivers discussed a balance between making the dispenser easy to use for a range of caregivers, while also ensuring the drugs are secure from children. Most caregivers and providers believed the responsibility was on the caregivers, not the manufacturer, to make the dispenser “child proof”; they explained how the medication should be in a locked box, or out of reach, of the children to ensure safe keeping.

*The packet should just remain as it is, so as a grown up you have been advised to keep it away from the child and that is how it should be. Caregivers to take full responsibility. (Hosp3_KII1_ID9)*

However, some also recommended that the dispenser have some child-proofing mechanism as a back up to secure storage.

*Aah, yes, we can make it child-proof by making it have some lockable way that only an adult person is able to, or a mature person is able to figure out and open. So, it should be designed in such a manner so that even if by mistake you forget it within the reach of the child, they are still not able it unless they are so crafty that they want to break it. (Hosp3_KII10)*

#### Size

In discussing size of the dispensers, participants weighed considerations related to portability and discretion to reduce stigma with its ability to accommodate various refill frequencies.

Caregivers and providers agreed that the dispenser should be small enough to facilitate easy carry and storage. A small dispenser was seen as essential for improved privacy/confidentiality, reduce stigma, and improve portability. Most providers discussed the ability to put the packaged ODS into bags; however, some provider mentioned that male caregivers, who do not carry purses, should also be able to carry the ODS in their pockets easily.

*The container should be small so that it can be carried in a small bag even for a week’s supply, or worse, four days. Also, it should be small such that even if I place it in the pocket, no one can notice it. Also, removing it for the children when I find them in public will not be hard (Hosp2_KII09)*.

A major consideration in discussing packaging size was the number of strips contained in the dispenser. In addition to carrying fewer doses while traveling, refill frequencies at the clinic varied based on the stability of the client and can range from 2 weeks to 3 months. Thus, dispensers would need to accommodate a different number of strips based on various patient care plan. To accommodate these considerations, some providers recommended having various dispenser sizes.

*I think, it would have, like two, sizes… two different sizes for… for if you want to administer medication for like a month, there is a smaller package, and if you want to umm to administer for like three months, there is also a package for those number of days. I am just thinking like that. So that if you have to give somebody for two months, you just give the one for one month, two of them. Yes. And for three months, instead of now giving three of them, you just give that one, that whole one for…for the three months. (Hosp2_KII8)*

Packaging that can accommodate various refill frequencies would also be beneficial in case of supply shortages, so that shorter refill appointments can be given to extend the supply.

*The packaging should be responsive in that manner such that even if we say we do packs of 30, how possible is it to separate and divide this in the event that you are having a shortage and so that you are not giving one person for one month and the other one is going without, so that you can give for two weeks each and within that time you are able to outsource for medicine and get more. (Hosp3_KII4)*

Emphasizing the challenges that some caregivers faced in accessing the clinic, caregivers and providers felt that asking the clients to return to the clinic more frequently to accommodate a smaller package size would be unacceptable. A majority felt they would prefer to have a larger dispenser, to decrease refill frequency. The caregivers discussed how they are used to carrying a large container for the current regimen, so a slightly larger dispenser would not bother them.

*I would take the sixty-one [instead of thirty] to avoid costs of travelling to the clinic all the time. Sometimes I do not have money to travel to the clinic often, so it is good if I take time to make money for my next visit. (Hosp3_FGD1_ID9)*

#### Material

Caregivers and providers discussed both preferred material for the dispenser, as well as the individual ODS wrapping.

Caregivers and providers emphasized the need for a dispenser that was light weight, durable, and did not become damaged or compromise the efficacy of the medication inside when exposed to water, heat, or humidity.

*It should be made waterproof (Hosp2_FGD1_ID6)*.

*If it falls in a hot surface, it should not interfere with the contents (Hosp2_FGD1_ID7)*

Caregivers were very optimistic about having a dispenser that is reusable to preclude the need to find a confidential way to dispose of single use drug containers. Stigma seemed to be a big factor in what drove the opinions behind the dispenser material. Caregivers expressed that they wanted a material that would decrease the risk of their peers automatically knowing what they were carrying: i.e., a material that would not make noise and a material other than plastic, a well-known drug container.

*Unlike the monthly drug bottles, that results to their pilling up, the re-usable dispensers will be superb. (Hosp1_FGD2_ID3)*

*It’s easy to carry. When you carry the metal one will be shaking too much ……., you know when you carry something like foil it will be easier for you and even when you take it out where people are they will not know what it is you are carrying. Plastic has been common that when you carry it someone will know you are carrying drugs with you. And if you change for us [to] the foil material, it would be good because one would mistake it for something else.* *(Hosp2_FGD2_ID4)*

Providers were split more evenly on preferences for metal vs. plastic containers. They felt that metal containers, while more durable, may become too hot to keep the drugs safe, is heavier than plastic and could be cumbersome to carry around, and could pose a danger to young children if they got their hands on it.

Key considerations for wrapper material included: easy disposal (ability to burn) to improve confidentiality and reduce stigma, portability, durability, and keeping the strips safe from the elements (including heat, humidity, rain) that were typical in the Kenyan context. The preferred material for individual packaging was plastic as this was seen as a secure, waterproof, heat-proof option that was easy to dispose of. Foil was a frequently mentioned alternative to plastic, however, concerns about how caregivers could confidentially dispose of the packets to reduce stigma if neighbors were to see them were raised since foil does not burn as easily.

#### Cost

Cost was discussed as a critically important element. Across the board, participants and providers felt that dispensers should be provided to patients free of cost. Given sociodemographic and financial constraints of many, providers felt that a charge to either the drugs or dispensers would severely hinder uptake and would result in nonadherence to refill appointments.

*my friend I’m telling you 80% of HIV clients are living below poverty level, these clients cannot afford to buy their own medication, like few months ago we had a shortage of prophylactic drugs, so we issued them free septrin, so if we do not dispense septrin, they will never buy. (Hosp3_KII6)*

In addition, providers felt that charging for ODS would damage the relationship between provider/hospital and patient, with the patient feeling that – since ARV are always free - the hospital is unjustly charging and profiting from the prescription of the drug.

*That [charging for ODS] will be very difficult because; most of our clients here know that services are offered for free. They might think that we staffs are using fraudulent tricks to charge them. (Hosp1_KII5)*

A few providers and caregivers conceded that – if explained thoroughly in advance – charging between 20 and 200 Kes (~ USD $0.20 - $2.00) for a replacement dispenser in cases that the original dispenser was mistreated and damaged would be OK; however, they still felt like this would hinder uptake and adherence, with patients delaying care after a broken dispenser if they were unable to afford a new one at that time.

*If they’ll be put on sale, then it will be good to consider designing durable dispensers that can last for a while. If the dispenser falls and break or get burnt accidentally….it should not cost above 200/=. (Hosp1_KII1)*.

*Charging for replacing will be a challenge… You know we are all abled differently. Some of us are struggling and don’t even know what they will eat, especially the HIV positive individuals living in the rural set ups. I can recommend that we quote a price of 50 shillings, but still, some cannot afford it.* *(Hosp1_FGD2_ID5)*

The primary recommendation to overcome challenges of cost was to create a durable, long-lasting dispenser that will not need to be replaced.

#### Other characteristics of packaging: color and shape

Neither dispenser color nor shape seemed very important to providers or caregivers.

In terms of color, many just stated their favorite color as the preferred color of the dispenser or mentioned that bright colors could be attractive and fun for children. Some suggested having cartoon characters or doodles on the dispenser to further attract children and enhance the experience. Some considerations that were discussed when thinking of dispenser colors were requirements of the drug, with a few providers stating that since drugs can be sensitive to light it should not be transparent. Others recommended a color-coding system where dispensers can be used as a discreet way to indicate the regimen or dosing of the strip contained within (e.g. blue for the smallest weight dose, green for the largest).

In terms of shape, a few caregivers indicated that having it shaped like a phone or tape recorder (which was present in the interviews and was used as a reference) would be acceptable and could improve confidentiality and decrease stigma. However, generally, participants did not have any strong opinions on shape if it was easy to carry in a pocket or purse.

## Discussion

Caregivers and providers expressed a strong desire for an easier way to administer medication, especially among children too young to swallow tablets whole, and expressed enthusiasm around the idea of ODS. Key preferences for ODS included a pleasant taste; one strip per dose with no need to measure or cut; small size with rapid dissolution; clear markings and instructions; and no special storage requirements. For packaging, stakeholders seemed to prefer individually wrapped strips within a dispenser. The individual packaging should be durable, waterproof, and easy to dispose of in communal spaces. They should also be easy to open, with clear serration and markings on where to open. The dispenser holding the strips should be durable, re-usable, accommodating of various refill frequencies but small enough to hold in a pocket or purse without others seeing it, and easy to use for children as young as 6.

The number of available pediatric ART formulations are limited compared to adult formulations [6]. Children only make up 7% of HIV cases, globally, and the recent focus on investing in PMTCT programs to reduce childhood infections creates a lack of incentive to invest in the development of child-friendly formulations. However, recent calls to scale up the global pediatric ART research agenda [21–23] has created space and incentives to accelerate the introduction of research to overcome known challenges to pediatric ART. Understanding user challenges with current formulations, as well as user preferences, is critical to design a product that will overcome current challenges and facilitate medication adherence [24]. Furthermore, as country guidelines start incorporating at-birth HIV testing for infants born to mothers with HIV, it becomes increasingly important that appropriate drug delivery systems be developed for even the youngest neonates.

Other novel formulations for pediatric ART are in the pipeline that offer promise to overcome some challenges of pediatric ART. LPV/r dissolvable pellets can be sprinkled on food or in drink, do not require cold storage, taste better than traditional formulations, and are generally more acceptable to caregivers than syrups or tablets; however, challenges still exist particularly in measuring the adequate dosage and in the number of steps required for administration (i.e. preparing food or drink, opening the capsule, measuring the pellets, mixing the pellets, administering food/drink to the child) [25]. Patches, implants, and injectables are also being investigated but these are in much earlier stages of development and being considered primarily for adolescents and adults [26]. ODS have the potential to be packaged in a “ready-to-use” solution, so caregivers can open the strip and administer to the child without additional preparation. If adequate taste masking can be achieved, this solution would overcome many of the barriers discussed.

Participants in this study expressed enthusiasm for ODS and how this novel drug delivery may help overcome challenges with pediatric ART. However, we acknowledge limitations of the study. Caregiver participants were actively engaged in their child’s care and the children they cared for were receiving ART. This population may not be representative of challenges and preferences of those less engaged in their child’s care and likely do not reflect the experiences of the approximately 40% of children living with HIV not receiving ART [4]. We also recognize that a myriad of caregiver sociodemographic characteristic including age and their own health, education, employment status, financial security, HIV disclosure status, and own adherence to ART can influence caregiver’s ability to provide ART care for their child, [8, 27] we were unable to purposefully select participants to represent all of the potential characteristics influencing pediatric ART adherence and, therefore, these results may not reflect those caregivers who have characteristics outside of what is reported in this study. Furthermore, caregivers of children less than 5 months of age were not represented in our results. The average age of ART initiation among perinatally infected infants is 17 weeks; [28] so while the range of caregivers represented in our study reflects this average, it is expected that guidelines recommending earlier and more streamlined testing will result in infants initiating ART earlier [13]. Lastly, while we selected facilities to represent a range of cultural and religious diversity within Kenya to increase generalizability of findings, the three hospitals selected are not representative of all facilities in Kenya.

### Conclusions and next steps

This study assessed user (caregiver and provider) preferences for oral dissolvable strips, a novel pediatric ART drug delivery system currently being developed. Implementation research with end-users beginning at the initial development phase is key to successful uptake and utilization of any new product, [17, 29–31] and it can avoid wasted investment in innovations that are not widely adopted. Our results indicated that user priorities for pediatric ART included: pleasant tasting, small size with rapid dissolution, easy to use without complex or time-consuming measuring or preparation. Priorities for packaging included: small, discrete, easy to carry packaging that offered protection from common environmental conditions (i.e. heat, humidity) without the need for special storage.

The results of this qualitative study were used to develop a range of ODS and packaging prototypes, with various sizes, flavors (orange, mango, strawberry, unflavored sweet), and packaging. These prototypes display a range of user preferences and will balance user preferred characteristics with feasibility constraints of developing the product. In the next phase of the study, focus groups with caregivers and providers will evaluate the prototypes developed based on the feedback from these interviews and focus groups. In addition, in this final phase of the study we will talk with children 6–10 years of age to assess their thoughts on the prototypes. These efforts will identify the leading ODS candidate. We will also identify compromises in preferences that are acceptable and unacceptable (e.g. would needing to administer two strips be acceptable if that meant a much smaller strip size, would a smaller/thicker strip be preferable over a larger/thinner strip that can contain the same drug volume). Concurrently, a Small Business Innovation Research grant (1R43AI170237) is underway to develop, evaluate, and optimize a an ODS containing three antiretroviral drugs designed specifically for pediatric ART.

### Electronic supplementary material

Below is the link to the electronic supplementary material.

Supplementary Material 1

Supplementary Material 2

Supplementary Material 3

Supplementary Material 4

## Data Availability

The datasets used and/or analysed during the current study are available from the corresponding author on reasonable request.

## List of abbreviations

ART: antiretroviral therapy
CCC: comprehensive care center
EID: early infant diagnosis of HIV
FGD: focus group discussion
HIV: human immunodeficiency syndrome
KII: key informant interview
MCH: maternal and child health
ODS: oral dissolvable strip
OPD: outpatient department
PMTCT: prevention of mother-to-child transmission of HIV
