# Supplementary material for: Assessing user preferences for design characteristics of oral dissolvable strips for pediatric HIV medication: a qualitative study
Source: BMC Health Serv Res. 2023 Oct 16;23:1103. doi: 10.1186/s12913-023-10078-6 (PMC10580521; doi:10.1186/s12913-023-10078-6)
Supplement: Supplementary file 1 — Supplementary Material 1 [file 12913_2023_10078_MOESM1_ESM.docx]

**Supplementary Material 1: Caregiver Focus Group Discussion Guide**

*Introductory text: Hello, and thank you for accepting to participate in this discussion. We will be here for about an hour. My name is _______________________. I will be leading the discussion today.*

*The purpose of this group discussion is to talk about issues related to ART medication for infants and children; specifically challenges with adherence and ways to reduce the challenges. Later in the discussion, I will introduce the concept of “oral dissolvable strips” – also called “ODS” - as a new way to delivery ART to infants and children and ask your opinions on them and what characteristics they should have to make them acceptable and easy to use. We will use this information to design ODS containing ART medication for infants and children.*

*I would like to let you know that we will be audio recording this discussion today. This recording will allow us to revisit the discussion later. This is a confidential discussion. As such, names of participants will not be collected or included in the final report about this meeting and I will not discuss what individual’s said with providers at this hospital or anyone else. We also ask that you do not discuss what your peers say in this group outside of this discussion.* *We stress confidentiality because we want an open discussion. We want all of you to feel free to comment on each other’s remarks without fear your comments will be repeated later and possibly taken out of context. We ask that each of you treat each member of this group with respect, giving each person a chance to speak and express their opinion freely.*

**Standard of Care Pediatric ART Administration**

*During the first part of our discussion, I’d like to learn about your experience providing ART to your child/children; including the process of administration, challenges, and motivators. As you share your experiences,* please identify which form of ART you are referring to in your response.

1. In what form does your child’s ART come in: tablets? Syrups? Other?
2. Are there any special storage requirements or considerations for the medication?
3. Can you please describe the process of giving your child his/her ART medication?

Probes

- 1. What steps do you take to prepare the medication (crush/reconstitute tablets? add sweeteners to improve the taste? shake syrups?). Could you role play how you prepare?
  2. How do you know how much medication (what dose) to give your child?
  3. How do you measure the dosage of medication to be given?
  4. How do you administer the medication to the child (via syringe? Cup? Whole tablet?)
  5. How confident do you feel that you are able to give your child their medication correctly each time.
  6. How many times per day do you give your child his/her ART medication?
  7. Do you administer and store the child’s ART in the same location?

1. What are some challenges associated with your child’s ART preparation and administration?

Probes

- 1. Do you administer ART medication to more that one child? If so, how do you manage the medication? How do you identify between each child’s medication? Are the children given different doses? How do you keep the medication syringes clean?
  2. How long does the preparation and administration take?
  3. Are you ever concerned that someone will see you preparing/administering the medication?
  4. Does the child willingly take the medication? If not, are there any times when your child does not get the full dose? (And, if so, how often does happen?) What are some things you try to do to help the child get the full dose?
  5. When you are not around, who gives the child his/her medication? How confident are you that others are able to give the child his/her medication correctly?
  6. What are the challenges you face when you are traveling? Is the medication convenient to carry with you? Does the child miss any doses when you are traveling?
  7. How often do you need to get the medication refilled? Is this frequency OK with you?

1. What are some things that help you give your child his/her medication?
   1. Are there specific things that help you remember to give your child his/her medication?
   2. Do you have any tip/tricks that simplify the preparation/administration of the child’s ART?
2. In thinking about medication design, are there any characteristics you think could be changed about your child’s current regimen to simplify giving your child his/her ART medication?

Probes:

- 1. For example, what could change about characteristics such as size of tablet, taste, shape, dosing frequency, method of administration to make pediatric ART easier on caregivers? Why?
  2. Which characteristic do you think is the most important to change?

**Introduction to ODS**

*Our team is working on developing an “oral dissolvable strip” – “ODS” for pediatric ART medications. Oral dissolvable strips are small, thin films that contain medication, adhere to the tongue/palate, dissolve in saliva or other liquid – releasing the medication.*

***[Show a sample ODS]***

*Our team has created these same strips for other medication delivery, but this will be the first attempt at making them for ART and – also – the first attempt at making them specifically for infants and young children.*

*Since they adhere to the mouth, they cannot be spit out. And since they dissolve quickly, they do not pose a choking risk – even to very young infants. Each segment contains a designated amount of drug. To administer to the infant/child, you measure the strip length indicated by their age or weight, adhere it to the child’s palate, and let it dissolve. Initiating breastfeeding or providing a beverage after administration can speed up the dissolution process. Flavorings and sweeteners can also be added to make them taste better.*

*Before I begin asking you questions regarding your perceptions of this way to deliver ART to children and your preferences, does anyone have any questions regarding the concept of ODS or how they are administered?*

1. What are your initial perceptions of ODS?
   1. How do you think ODS would affect your experience giving ART to your infant/child? Probe: Would they make administering ART to your infant/child easier or harder? Why do you think this? How about giving maximum dose and the thickness of multiple strips in the mouth?
   2. What concerns do you have about using them for infant/child ART medications?
   3. Assuming equal effectiveness, if given the option, do you think you would try ODS over your infant/child’s current regimen – why or why not?
2. What do you think are important characteristics about ODS that should be considered to support pediatric ART adherence?

Probes:

Do you think the following are important? If so, what are your preferences for these characteristics?

Do you think the following are important? If so, what are your preferences for these characteristics?

- 1. Size & shape – Do you have any preferences for the size and shape of the ODS? What dimensions would be best?
  2. Strip color: do you have any preferred color?
  3. Strip markings: We could label the strips with including weight or age bands to indicate dose per length of strip, medication name, etc. Would you like any such markings to be added to the strips? Why or why not? How big would you imagine the markings be? Would it need to be high in contrast?
  4. Taste: What are some important taste considerations?
  5. Dosing frequency: Ideally, we believe we can design these to support once-daily dosing. What are your thoughts on this dosing schedule? Would more frequent dosing be acceptable, if necessary?
  6. Dissolution: how quickly do you think these need to dissolve to be acceptable?
  7. Time for administration: Do you think the process of administering this medication will be quicker or slower than the conventional method when you use ODS? How important is it for you to be able to do this quickly?
  8. Any other characteristics you think are important?

1. Which of these characteristics do you think are MOST important? Which are LEAST important? Why?
2. What characteristics would dissuade you from trying ODS? What characteristics would make you want to try ODS? Why?
3. What characteristics would make adherence more difficult? What characteristics would make adherence easier? Why?
4. What other things do you think need to be considered in the design and development of ODS for infant/child ART medication?

**Dispenser characteristics**

*In addition to designing the strip, we need to also develop an “ODS dispenser” – that is a container that will hold and help allocate the strips. Like the strip, we want to make sure that dispenser design supports ease of use for caregivers. There are several ways that a dispenser could be designed: different sizes, colors, methods of distribution are available.*

***[show a few example designs and talk about each one]***


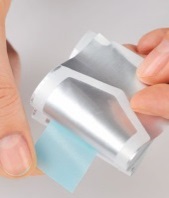


C


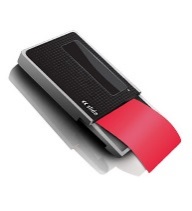


b


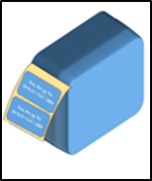

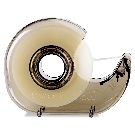


a

- *ODS could come in a roll, with serrated dosing marks and a dispenser that holds them all – a bit like a tape dispenser (Option A)*
- *Dispensers could be just a few centimeters and distribute single strips at a time. While convenient, this may make it more difficult if your infant/child is not of the age/weight to require a whole strip.* (Option B)
- *Rather than design a dispenser, strips could be individually wrapped in foil. Like single strip dispensers, this would require caregivers to use portions of a strip if the child was not at the age/weight to use the entire strip (Option C)*

1. What are some of the things that you like about each of these designs? What are some of the things you don’t like?
2. What are some important considerations regarding each of the following characteristics?
   1. Size – What is size range that you think would be ideal for a dispenser? Why?
   2. Refill frequency
   3. Dosing – separate individual strips or tape-like strips, which do you think would be preferable? Why?
   4. Shape
   5. Color
   6. Dispenser material: plastic? Metal? Foil packets? Why?
   7. Other characteristics?
3. Do you have any concerns with the child getting into the medication by him/herself? To what degree would you like the packaging to be childproofed? Why? How could the dispenser be designed to look hazardous to the child and not enticing?
4. Which of these characteristics do you think is MOST important, if we weren’t able to accommodate them all? For example, if a smaller dispenser size would not accommodate a less frequent refill frequency, would you rather compromise on size or frequency?

**Group Activity**

*For this next portion of the discussion, I’d like you to form three groups. I will give each group a flipchart and some markers. Given your groups’ preferences for ODS and dispenser, I’d like to ask you to draw your ideal ODS and dispenser. I will give you 10-15 minutes to do so. After, I will give each group a time to talk about their designs and why they chose those particular characteristics.*

*[****Allow each group to work, reconvene once groups seem to be done. Allow each group to discuss their diagrams & ask probing questions about things that may not be displayed in their picture (i.e. taste, size (if not to scale), etc.****]*

1. Before we wrap up, who has concluding remarks that they’d like to add to this discussion?
