# Supplementary material for: Assessing user preferences for design characteristics of oral dissolvable strips for pediatric HIV medication: a qualitative study
Source: BMC Health Serv Res. 2023 Oct 16;23:1103. doi: 10.1186/s12913-023-10078-6 (PMC10580521; doi:10.1186/s12913-023-10078-6)
Supplement: Supplementary file 2 — Supplementary Material 2 [file 12913_2023_10078_MOESM2_ESM.docx]

Supplementary Material 2: Provider Interview Guide

*Introductory text: Hello, and thank you for accepting to participate in this discussion. We will be here for about an hour. My name is _______________________.*

*The purpose of this group discussion is to talk about issues related to pediatric ART medication; specifically challenges with adherence and ways to reduce the challenges. Later in the discussion, I will introduce the concept of “oral dissolvable strips” – also called “ODS” - as alternate formulation for pediatric ART and ask you your opinions on them and what characteristics they should have. We will use this information to design ODS containing ART medication for infants and children.*

*I would like to let you know that we will be audio recording this discussion today. This recording will allow us to revisit the discussion later. This is a confidential discussion.*

1. Can you tell me about some challenges that caregivers face in giving their infants and children (<10 years) ART? (Probes: Related to accessing medication/attending clinic? Related to remembering to give ART? Related to preparing/administering ART?)
2. What is the most important challenge you think caregivers face in preparing and administering ART to their children?
3. Can you tell me about some challenges that you, as a [provider role] face in the provision of pediatric ART?

Probes: Supplies/stock outs; storage; coordination between departments; late patient presentation for care; adherence/retention; etc.?

**Introduction to ODS**

*Our team is working on developing an “oral dissolvable strip” – “ODS” for pediatric ART medications. Oral dissolvable strips are small, thin films that contain medication, adhere to the tongue/palate, dissolve in saliva or other liquid – releasing the medication.*

***[Show a sample ODS]***

*Our team has created these same strips for other medication delivery, but this will be the first attempt at making them for ART and – also – the first attempt at making them specifically for infants and young children.*

*Since they adhere to the mouth, they cannot be spit out. And since they dissolve quickly, they do not pose a choking risk – even to very young infants. Each segment contains a designated amount of drug. Thus, to administer to the infant/child, you measure the strip length indicated by their age or weight, adhere it to the child’s palate, and let it dissolve. Initiating breastfeeding or providing a beverage after administration can speed up the dissolution process. Flavorings and sweeteners can also be added to make them taste better.*

*Before I begin asking you questions regarding your perceptions of this drug administration route and preferences, do you have any questions regarding the concept of ODS or how they are administered?*

1. What are your initial perceptions of ODS?
   1. What concerns do you have about using them for infant/child ART medications?
   2. If given the option, do you think caregivers would try them over current regimens – why or why not?
   3. If given the option, do you think providers would recommend them over current regimens – why or why not?
2. What are important characteristics about ODS that should be considered to support prescription of ODS at the facility level and pediatric ART adherence at the individual level?

Probes: What are some important considerations regarding each of the following characteristics?

1. Size & shape – what dimensions would it need to be? Is there an acceptable range?
2. Strip color: do you have any preferred color, would that be important at all?
3. Strip markings: We could label the strips with including weight or age bands to indicate dose per length of strip, medication name, etc. Would you like any such markings to be added to the strips? Why or why not?
4. Taste: What are some important taste considerations? Are there any flavors you think would be most popular among caregivers and their children?
5. Dosing frequency:
   1. Ideally, we believe we can design these to support once-daily dosing.
   2. What are your thoughts on this dosing schedule?
   3. Would more frequent dosing (2x daily) be acceptable, if necessary?
6. Dissolution: How quickly do you think these need to dissolve to be acceptable?
7. Shelf-stability and storage considerations:
   1. With what frequency do patients receive ART refills?
   2. How long are ART stored at the hospital prior to distribution to patients?
   3. How are ART stored at the hospital? At the patients home?
8. Adherence measurements:
   1. How pediatric adherence to ART is typically measured?
   2. How can ODS be designed to accommodate this?
9. Any other characteristics you think are important? Instructions for Use? Labeling? Sterility?
10. Which of these characteristics do you think are MOST important? Which are LEAST important? Why?
11. What characteristics would dissuade you from adopting ODS at the facility level? What characteristics would encourage uptake?
12. What characteristics would make adherence more difficult at the individual level? What characteristics would make individual adherence easier?
13. What other things do you think need to be considered in the design and development of ODS for infant/child ART medication?

**Dispenser characteristics**

*In addition to designing the strip, we need to also develop an “ODS dispenser” – that is a container that will hold and help allocate the strips. Like the strip, we want to make sure that dispenser design supports ease of use for caregivers. There are several ways that a dispenser could be designed: different sizes, colors, methods of distribution are available.*

***[show a few example designs and talk about each one]***


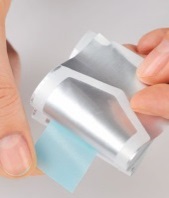


C


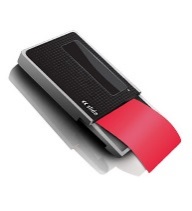


b


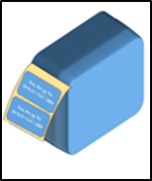

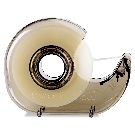


a

1. *ODS could come in a roll, with serrated dosing marks and a dispenser that holds them all – a bit like a tape dispenser*
2. *Dispensers could be just a few centimeters and distribute single strips at a time. While convenient, this may make it more difficult if your infant/child is not of the age/weight to require a whole strip.*
3. *Rather than design a dispenser, strips could be individually wrapped in foil. Like single strip dispensers, this would require caregivers to use portions of a strip if the child was not at the age/weight to use the entire strip*
4. What are some characteristics of each of the previously mentioned options that you like and don’t like? What are strengths and weaknesses of each design?
5. What are some important considerations regarding each of the following characteristics?
6. Size – What is size range that you think would be ideal for a dispenser? Why?
7. Refill frequency
8. Dosing – separate individual strips or tape-like strips, which do you think would be preferable? Why?
9. Shape
10. Color
11. Dispenser material: plastic? Metal? Foil packets? Heat and/or Transporting Impacts?
12. Cost of ODS/dispenser: What is an acceptable price point to support facility level adoption?
13. Other characteristics?
14. Which of these characteristics do you think is MOST important, if we weren’t able to accommodate them all? Which are LEAST important? Why? (For example, if a smaller dispenser size may not accommodate a less frequent refill frequency, would you rather compromise on size or frequency?)
