# Supplementary material for: Assessing user preferences for design characteristics of oral dissolvable strips for pediatric HIV medication: a qualitative study
Source: BMC Health Serv Res. 2023 Oct 16;23:1103. doi: 10.1186/s12913-023-10078-6 (PMC10580521; doi:10.1186/s12913-023-10078-6)
Supplement: Supplementary file 3 — Supplementary Material 3 [file 12913_2023_10078_MOESM3_ESM.docx]

**Supplementary Material 3: Caregivers and Provider Code Tree**

1. Standard of care Pediatric ART
   1. Formulation type
   2. Storage
   3. Preparation/measuring/administration method
   4. Frequency and Timing
2. Facilitators to SOC Administration
   1. Child cooperation
   2. Bribes
   3. Threats
   4. Cues to action
3. Challenges with SOC administration
   1. Individual-level challenges
      1. Forgetfulness
      2. Privacy/confidentiality
      3. Reliance on other caregivers
      4. Child anxiety over medication
      5. Child refusal of medication
   2. Community level
      1. Privacy/confidentiality
      2. Stigma
   3. Regimen related challenges
      1. Measuring/dosing
      2. Spillage
      3. Bulkiness of syrups
      4. Taste
      5. Side effects
      6. Complexity
      7. Timing/frequency of dosing
   4. Environmental/System level considerations
      1. Frequency of ART refill
      2. Patient retention*
      3. Supply stockouts*
      4. Provider workload*
      5. Coordination between departments*
4. Suggestions to improve standard of care
   1. Timing/frequency
   2. Formulation type
   3. Tablet size
   4. Smell
   5. Taste
5. Perceptions of ODS
   1. Perceived benefits of ODS
   2. Concerns with ODS
   3. Perceived acceptability of ODS
6. Preferred characteristics of ODS
   1. Size and shape
   2. Color
   3. Markings
   4. Taste
   5. Dosing frequency
   6. Dissolution
   7. Storage/shelf stability
   8. Adherence measurements*
   9. Other
7. Preferred characteristics of dispensers
   1. Size
   2. Shape
   3. Color
   4. Material
   5. Cost
   6. Single vs multi-serve
   7. Refill frequency
   8. Sounds

*Probed for providers only
